# Rates of breastfeeding initiation and duration in the United States: data insights from the 2016–2019 Pregnancy Risk Assessment Monitoring System

**DOI:** 10.3389/fpubh.2023.1256432

**Published:** 2023-12-19

**Authors:** Laura E. Diaz, Lynn M. Yee, Joe Feinglass

**Affiliations:** ^1^Department of Obstetrics and Gynecology, Northwestern University Feinberg School of Medicine, Chicago, IL, United States; ^2^Division of General Internal Medicine, Northwestern University Feinberg School of Medicine, Chicago, IL, United States

**Keywords:** breastfeeding, Pregnancy Risk Assessment Monitoring System, birth outcomes, pregnancy, breastfeeding benefits

## Abstract

**Introduction:**

While breastfeeding rates in the United States have been increasing, they remain low by international standards with substantial racial, income and education disparities. This study uses recent population-based data to analyze sociodemographic differences in breastfeeding initiation, duration, and exposure to information and education.

**Methods:**

We used the 2016–2019 Pregnancy Risk Assessment Monitoring System (PRAMS) to compare breastfeeding duration among a representative population from 43 states and the District of Columbia. We modeled the likelihood of never initiating breastfeeding by respondent’s age, race and ethnicity, language, marital status, household income, educational attainment, parity and insurance status. We also compared sources of information and education for respondents who never breastfed to those who breastfed up to 6 months.

**Results:**

Among 142,643 new mother respondents, representing an estimated population of 7,426,725 birthing individuals, 12.6% never breastfed, 60.4% reported breastfeeding at 3 months and 54.7% at 6 months. While 75.8% of college graduates reported breastfeeding at 3 months, this was only 37.8% of respondents with high school or less. Among those with the lowest six-month rates were non-Hispanic Black participants (36.3%) and those age < 20 (25.5%). Respondents with Medicaid coverage for their delivery were 25% more likely to have never breastfed than the privately insured. Respondents reporting household income <$20,000 were 57% more likely to have never breastfed as compared to those with household income>$85,000. While 64.1% of those breastfeeding at 6 months reported receiving information from “my” doctor’, this was only 13.0% of those who never breastfeed.

**Discussion:**

Improved breastfeeding rates could have significant effects on reducing health disparities in the United States. Clinical and public health policy initiatives need to include culturally sensitive breastfeeding education before and after childbirth, with psychological and direct support from obstetrics and primary care providers. Health plans should support home and community-based in-person and telelactation consulting services. Public policies such as paid family and medical leave and workplace accommodations will also be critical. Given the huge implications of breastfeeding rates on the development of infant immune defenses and a healthy microbiome, improving breastfeeding rates should be a much more important public health priority in the United States.

## Introduction

Breastfeeding is considered the gold standard for infant feeding and nutrition by the American Academy of Pediatrics with many documented benefits for both infants and their birthing parents (1). Benefits for infants include reduced risk of Sudden Infant Death Syndrome, asthma, ear infections, childhood obesity, gastrointestinal infections, and necrotizing enterocolitis for preterm infants (1, 2). Breast milk has the ideal amount of fat, sugar, water, protein, and minerals needed for infants’ growth and development (2) contains protective antibodies and is easier to digest than formula (3). Maternal benefits include decreased risk of hypertension, type 2 diabetes, ovarian cancer, and breast cancer (2). Breastfeeding also releases oxytocin, a hormone that causes the uterus to contract. This helps return the uterus to return to normal size more quickly and reduces bleeding after birth (3). Evidence suggests that women who planned to breastfeed and went on to do so were about 50% less likely to become depressed than mothers who had not planned to, and, did not breastfeed (4). A 2007 analysis found that if 90% of U.S. families breastfed exclusively for 6 months, it would prevent an excess of 911 preventable infant deaths per year (5).

Although rates of breastfeeding in the United States have improved significantly in the last 50 years (6, 7), only one in four infants are exclusively breastfed for 6 months (1). In 2018, only 25.8% of infants were breastfed exclusively for 6 months and 35.9% were breastfed to any extent at 1 year (8). A remarkable 60% of mothers stop breastfeeding sooner than they planned (1). Although the rate of ever breastfeeding for Black infants has increased significantly from less than 60% in 2007 (9) to 74%, this still lags the national average of 83% (1).

Sharply lower rates of breastfeeding among low income and minority mothers has major implications for the generation of lifecourse health disparities. The immunological and nutritional benefits of breastfeeding have been shown to reduce chronic inflammation, and thus cardiovascular disease, both directly and indirectly through reduction of obesity (10–13). Conversely, gut microbiome studies have shown the obesogenic effects of formula feeding (14).

This study was undertaken to better understand the challenges to increasing the likelihood of breastfeeding in the United States. We aimed to analyze sociodemographic differences in breastfeeding initiation (breastfeeding at all) and duration (breastfeeding at 3 months or at time of survey or 6 months or at the time of survey). We present data from the Pregnancy Risk Assessment Monitoring System (PRAMS), which provides population-weighted data from forty-three states and the District of Columbia (DC). Our first study aim was to present rates of breastfeeding discontinuation by respondent sociodemographic and clinical characteristics. Our second aim was to model the likelihood of no breastfeeding initiation after delivery, using the same respondent characteristics. Our third aim was to analyze differences in respondents’ reported exposure to breastfeeding information and education, comparing those who breastfed at 6 months versus those who never initiated breastfeeding. These findings are discussed in the context of efforts to increase breastfeeding rates for vulnerable populations.

## Methods

### PRAMS study sample

This study uses population-weighted data from 2016 to 2019 from the Pregnancy Risk Assessment Monitoring System (PRAMS) (15). PRAMS collects information about behaviors and experiences before, during, and after delivery of people who have been pregnant. All responses were obtained after informed consent by mail or with telephone follow-up for initial non-responders. This initiative is supported by the Centers for Disease Control and Prevention’s (CDC) Division of Reproductive Health. Participants were contacted via mail, telephone or both modalities to complete surveys within 2 to 6 months after delivery (15). The survey responses are linked to birth certificate data. This study sample included birthing individuals in forty-three states and the District of Columbia (DC). Arizona, California, Idaho, Nevada, Ohio, South Carolina, and Texas were not included in the PRAMS dataset. PRAMS sets the required response rate for participating states at 55% and in 2014 the median response rate was 61% (15). Our analyzes included all respondents with singleton, live births and without missing data for breastfeeding or sociodemographic characteristics, excluding 6.3% of respondents. PRAMS data are de-identified and publicly available and therefore IRB exempt at our institution. We followed STROBE guidelines for observational studies.

### Social demographic characteristics

Study variables were derived from both the PRAMS respondent survey and linked birth certificate data. Maternal age was categorized as less than 19, 20 to 24 years old, 25 to 29 years old, 30 to 34 years old, 35 to 39 years old, and greater than 40 years old. Maternal race and ethnicity categories included non-Hispanic white, non-Hispanic Black, Asian, Hispanic, and other or missing race and ethnicity. Maternal household income was categorized as greater than $85,00, $60,001-85,000, $40,001-60,000, $20,001-40,000, and less than $20,000. Household income level was regression-imputed for 7,234 (4.45%) respondents with missing data using respondent age, race and ethnicity, marital status, and education. Maternal educational attainment was categorized as less than 12 years of education, 12 years of education, 13 to 15 years of education and more than 16 years of education. The number of previous live births (parity) was categorized as 0, 1, 2, or three or greater. Other variables included if respondents were married, if the PRAMS survey was completed in Spanish, if they lacked health insurance coverage pre-conception, or if they had Medicaid coverage (versus any other insurance coverage) at delivery.

### Breastfeeding variables

Initiation of breastfeeding was determined by asking respondents if they ever breastfed. If respondents answered “no” it was categorized as never breastfed. Duration of breastfeeding was determined by asking respondents how many weeks or months they breastfed and if they were currently breastfeeding at the time of survey. The initiation and duration of breastfeeding were sorted into four categories based on the responses, ever breastfed, never breastfed, if respondents were breastfeeding at 3 months or time of survey and breastfeeding at 6 months or time of survey.

### Sources of information and support for breastfeeding

PRAMS respondents were asked whether a health care worker had asked them about whether they planned to breastfeed. They were also asked ‘Before or after your new baby was born, did you receive information about breastfeeding from any of the following sources?’ The sources included doctor, nurse (midwife, doula), breastfeeding or lactation specialist, baby’s doctor or health care provider, breastfeeding support group, breastfeeding hotline (or toll-free number), family or friends, or another source. To better assess the potential importance of each type of messaging, responses were compared by whether respondents ever initiated breastfeeding or were breastfeeding at 6 months or time of survey.

### Statistical analysis

Chi square tests were used to determine the significance of bivariate differences between respondent characteristics and whether respondents reported breastfeeding at each interval and for the significance of differences in the information and support responses between those who never initiated breastfeeding and those who were breastfeeding at 6 months. Logistic regression was used to test the significance of respondent characteristics for the likelihood of ever breastfeeding. All results are reported as population-weighted estimates using the complex survey module of Stata Version 16 (College Station, TX).

## Results

### Sociodemographic characteristics

The study sample included 142,643 PRAMS respondents with live singleton births and non-missing study data between 2016 and 2019. The weighted study cohort represented an estimated 7,426,725 birthing individuals in forty-three states and DC. While 12.6% of the sample population never breastfed, 87.4% initiated breastfeeding, 60.4% reported breastfeeding at 3 months or time of survey, and 54.7% at 3 months or time of survey.

Table 1 presents sociodemographic characteristics of PRAMS respondents by duration of breastfeeding. All comparisons were significant across each duration of breastfeeding intervals (*p* < 0.001) except trend in breastfeeding by survey year and Spanish language. Respondents ages 19 or younger, although only 4.2% of the sample, were the least likely to initiate breastfeeding with higher never breastfed rates continuing through age 24 and then dropping among older respondents. Older respondents were over 25% more likely to have continued to breastfeed at 6 months or time of survey than those age 24 or younger. Non-Hispanic Black respondents had the highest percentage of never breastfeeding (22.1%) while Hispanic, Asian, and other race and ethnicity respondents had the lowest percentage of never breastfeeding. Only 36% of Non-Hispanic Black respondents were breastfeeding at 6 months or at the time of the survey compared to Non-Hispanic White (59.2%), Hispanic (51.0%) and Asian (69.0%) respondents.

**Table 1:** Breastfeeding duration for respondents with live, singleton births in the 2016–2019 Pregnancy Risk Assessment Monitoring System, 43 states and the District of Columbia.^a^

	Sample	Row percent never breastfed	Row percent ever breastfed	Row percent breastfeeding at 3 months or time of survey	Row percent breastfeeding at 6 months or time of survey
Population-weighted *N*^a^	7,426,725	932,056	6,494,668	4,490,970	3,359,966
Sample percent	100	12.6	87.4	60.4	54.7
Survey year					
2016	20.3	12.7	87.3	61.0	55.2
2017	24.0	12.6	87.4	60.4	54.5
2018	27.2	12.6	87.5	60.2	54.4
2019	28.5	1.2	87.6	60.5	55.0
Age group					
<20	4.2	21.5	78.5	32.7	25.5
20–24	18.3	16.8	83.2	46.0	39.0
25–29	29.2	12.7	87.3	59.9	53.9
30–34	29.9	9.8	90.1	68.8	64.1
35–39	15.1	10.1	89.9	69.0	64.0
>40	3.3	11.4	88.6	65.9	60.7
Race and ethnicity					
Non-Hispanic White	59.2	11.8	88.2	63.4	59.2
Non-Hispanic Black	14.8	22.1	77.8	44.7	36.3
Hispanic	16.3	8.6	91.3	59.2	51.0
Asian	5.3	7.5	92.5	74.1	69.0
Other	4.4	10.8	89.2	62.0	55.0
Spanish preferred language	11.6	8.5	91.5	62.0	53.7
Legally married	62.7	8.0	92	71.4	66.8
Household income					
< $20,000	24.0	22.5	77.5	40.5	33.5
$20,001–$40,000	21.5	14.5	85.5	53.6	46.7
$40,001–$60,000	13.7	9.6	90.3	65.2	59.2
$60,001–$85,000	11.3	8.4	91.6	68.2	63.3
<$85,000	29.4	5.9	94.1	76.7	72.7
Educational attainment					
Less than 12 years	22.0	22.6	77.4	44.6	37.8
12 years	32.6	2.0	80	44.5	37.5
13 to 15 years	25.1	12.4	87.6	56.3	49.7
16 or more years	20.2	4.7	95.3	78.7	75.8
Parity					
0	39.0	9.6	90.4	59.7	54.0
1	33.1	12.7	87.3	62.2	56.7
2	16.4	15.3	84.7	60	54.5
>3	11.5	18.3	81.8	58.6	52.1
Uninsured pre-conception	15.6	14.1	85.9	55.3	48.5
Medicaid at delivery	24.3	22.0	78.0	42.6	35.3

a Includes respondents in 43 states and the District of Columbia in the 2016–2019 Pregnancy Risk Assessment Monitoring System; *n* = 142,643 respondents with live singleton births from forty-three states and the District of Columbia, excluding Arizona, California, Idaho, Nevada, Ohio, South Carolina, and Texas. Population weighted *n* = 7,426,725. All comparisons across intervals significant at *p* < 0.001 except for Spanish preferred language and year of birth.

**Figure 1:**
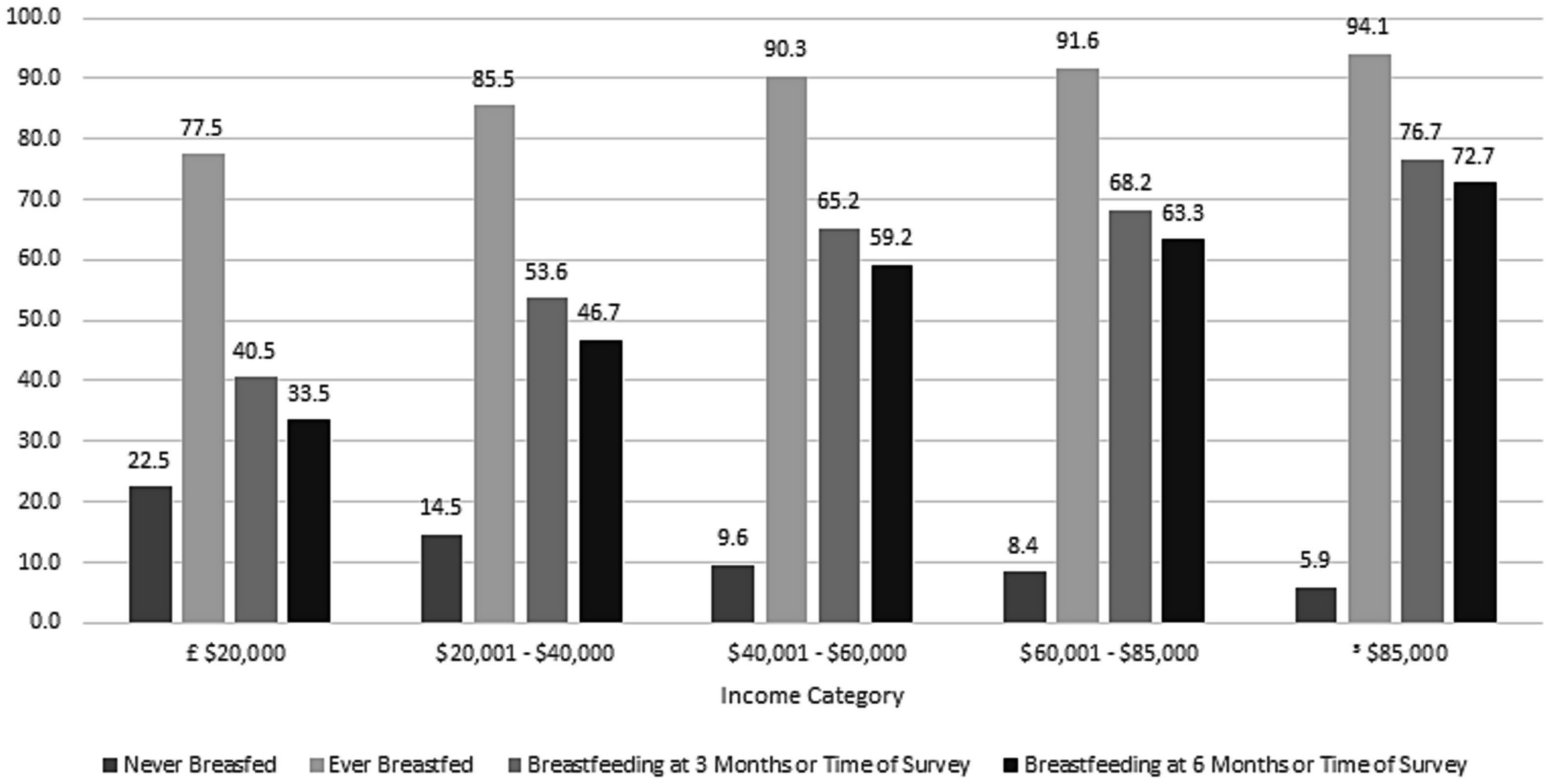
Differences in breastfeeding initiation and duration by household income. 2016–2019 Pregnancy Risk Monitoring System 43 states and the District of Columbia, weighted *n* = 7,426,725.

**Figure 2:**
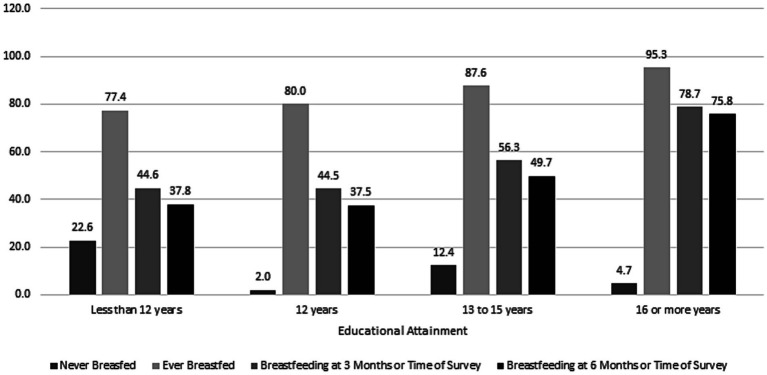
Differences in breastfeeding initiation and duration by maternal education. 2016–2019 Pregnancy Risk Monitoring System 43 states and the District of Columbia, weighted *n* = 7,426,725.

There was a gradient of breastfeeding rates across household income categories (Table 1; Figure 1). Among respondents reporting an annual household income of less than $20,000, 22.5% never breastfed as compared to only 5.9% of those with a household income over $85,000. Similarly, among respondents reporting an education level of high school or less, 33.5% were breastfeeding compared to 75.8% of respondents at highest level of education at 6 months or time of survey (Figure 2). Respondents who were uninsured before pregnancy and those with Medicaid delivery coverage had much lower rates of breastfeeding initiation and duration (Table 1).

### Modeling the likelihood of never initiating breastfeeding

Table 2 presents logistic regression odds ratios and 95% confidence intervals for the likelihood of never breastfeeding. There was a modest but significant decrease in never breastfeeding after 2016. As compared to the reference category of respondents ages 25–29, respondents over age 35 were increasingly less likely to have ever initiated breastfeeding. Non-Hispanic Black respondents are 12% more likely to have never breastfed while other racial and ethnic groups were significantly less likely to have never initiated breastfeeding as compared to Non-Hispanic White respondents. Respondents with a household income of <$20,000 were 57% more likely to never breastfeed as compared to those with an income greater than $85,000, but differences by income become non-significant for those above $40,000. In contrast, the education gradient was steeper, with the least educated individuals over four times more likely to have never initiated breastfeeding. Nulliparous respondents were much less likely to never initiate breastfeeding, as parity increased the likelihood of never breastfeeding increased. Respondents with Medicaid coverage were 25% more likely to have never initiated breastfeeding.

**Table 2:** Logistic regression results for the likelihood of never breastfeeding. Pregnancy Risk Assessment Monitoring System, 43 states and the District of Columbia.

	Odds ratio	95% confidence interval
Survey year		
2016	Reference	
2017	0.92	(0.85–0.99)
2018	0.89	(0.83–0.96)
2019	0.89	(0.83–0.96)
Age group		
≤19	0.99	(0.87–1.31)
20–24	0.96	(0.89–1.04)
25–29	Reference	
30–34	1.06	(0.98–1.14)
35–39	1.18	(1.08–1.29)
≥40	1.29	(1.08–1.29)
Race and ethnicity		
Non-Hispanic White	Reference	
Non-Hispanic Black	1.12	(1.05–1.20)
Hispanic	0.46	(0.42–0.51)
Non-Hispanic Asian	0.84	(0.74–0.96)
Other	0.60	(0.53–0.67)
Spanish preferred language	0.67	(0.57–0.77)
Legally married	0.61	(0.57–0.65)
Household income		
≤$20,000	1.57	(1.42–1.74)
$20,001–$40,000	1.19	(1.07–1.31)
$40,001–$60,000	1.04	(0.94–1.16)
$60,001–$85,000	1.06	(0.94–1.19)
≥$85,000	Reference	
Educational attainment		
Less than 12 years	4.33	(3.87–4.84)
12 years	3.15	(2.86–3.47)
13 to 15 years	2.02	(1.84–2.21)
16 or more years	Reference	
Parity		
0	Reference	
1	1.40	(1.35–1.55)
2	1.54	(1.42–1.68)
≥3	1.54	(1.35–1.55)
Uninsured pre-conception	0.95	(0.87–1.04)
Medicaid at delivery	1.25	(1.17–1.34)

Includes female respondents in 43 states and the District of Columbia in the 2016–2019 Pregnancy Risk Assessment Monitoring System; *n* = 142,643 respondents with live singleton births from and the District of Columbia, excluding Arizona, California, Idaho, Nevada, Ohio, South Carolina, and Texas. Population weighted *n* = 7,426,725.

### Sources of breastfeeding information

**Figure 3:**
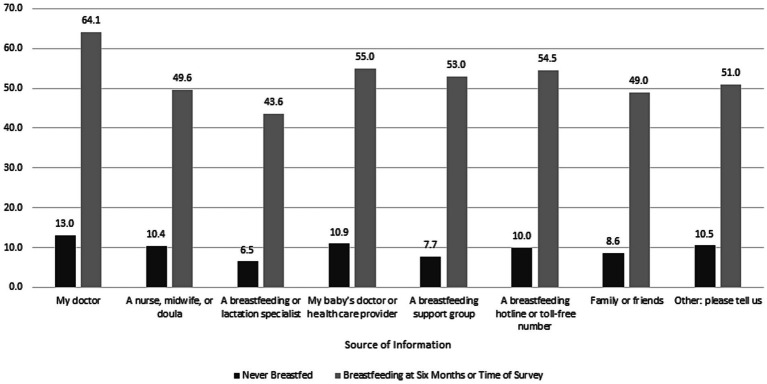
Differences in sources of breastfeeding information and support between respondents who breastfed for 6 months or time of survey versus respondents who never initiated breastfeeding, 2016–2019 Pregnancy Risk Monitoring System, 43 states and the District of Columbia weighted *n* = 7,426,725.

Figure 3 presents information about who provided breastfeeding information and support, showing differences between those who never initiated and those who were breastfeeding at 6 months or time of survey. Only 7.7% of respondents reported that a health care worker had asked about whether they planned to breastfeed. The figure shows that among the 12.6% of respondents who never breastfed, 10% or less received information or support from anyone, including only 8.6% from ‘family and friends. Among the 54.7% reporting breastfeeding at 6 months or time of survey, 64.1% received information from their doctor and 43.6% from a breastfeeding or lactation specialist as compared to only 8.6% of respondents who never breastfed.

## Discussion

Our findings point to large income, education and racial disparities in breastfeeding initiation and duration that need to be the focus of major public health and clinical policy initiatives. Prenatal and postpartum obstetric care offers significant opportunities for improving breastfeeding education, with peer counseling support through the postpartum period providing a particularly effective approach (16). The US Preventive Services Task Force also recommended that primary care clinicians should support women before and after childbirth to help them make an informed choice about how to feed their infants (17, 18). These professional support, peer support, and formal education interventions can include promoting the benefits of breastfeeding, providing practical advice and direct support on how to breastfeed, and providing psychological support (17). A systemic review found that breastfeeding support was associated with a 16% increased likelihood of exclusive breastfeeding at 6 months (17, 19). The American College of Obstetricians and Gynecologists (ACOG) recommends a multidisciplinary approach involving community, family, parents, and health care professionals to strengthen the support for parents and help them achieve their breastfeeding goals (2).

### Policies for achieving higher breastfeeding rates in under-resourced communities

State Medicaid plans, which cover 42% of all births in the U.S., affect breastfeeding support services for low-income women. Lactation consultation services are more commonly covered in the hospital setting, but having coverage for lactation consultants and breast pumps (which since the Affordable Care Act are available yearly), in outpatient or home settings has been shown to increase breastfeeding (20, 21). Maximizing the expansion of telehealth services could bring breastfeeding specialists into communities that lack them. For instance, telelactation services that connect lactation consultants with breastfeeding parents through video visits could reach hundreds of thousands of families each year (19). The effects could be enhanced by allowing parents to choose a lactation consultant with a similar cultural background (19). However, this option is limited for those living without internet access. Medicaid coverage of Doula care can also provide a cost effective intervention (22–24).

Extended paid maternity leave benefits, which are ubiquitous in other wealthy countries but only exist in a handful of U.S. states, have also been shown to increase initiation and duration of breastfeeding among working class families (25–29). The Women Infant and Children (WIC) program, with 1800 local agencies, is uniquely positioned to engage vulnerable populations in providing breastfeeding support. Many WIC agencies are successful at providing peer breastfeeding support. While less than 40% of all state and local WIC agencies provide clients with lactation support services from a Board-Certified lactation consultant, agencies can make referrals to local hospitals or regional lactration consultants (30). Workplace interventions such as breaktime for nursing mothers (31, 32), home visiting programs (33, 34), and community organizations such as La Leche League (35) and the Erikson Institute Fussy Baby Network (36) can also play an important role in increasing breastfeeding rates. Finally, obstetric providers need to redesign prenatal care to better regulate the marketing of breast milk substitutes and enhance prenatal education and postpartum breastfeeding support. This approach is part of the Baby-Friendly Hospital Initiative (37–40).

### Limitations

PRAMS data do not include respondents from large states such as Texas, California and Ohio, and are less than nationally representative. PRAMS breastfeeding duration questions can only provide rough estimates in so far as interviews are conducted at varying time intervals from 2 to 6 months after birth. These questions also did not include whether breastfeeding was exclusive. Finally, publicly available national PRAMS data do not provide respondents’ reasons for not initiating or curtailing breastfeeding, a crucial area for future qualitative research.

## Conclusion

Increasing breastfeeding rates would have major implications for reducing socioeconomic and racial disparities, including obesity, in the United States population (10, 41, 42). Breastfeeding effects on the development of infant immune defenses and a healthy microbiome are closely related to subsequent cardiovascular health (43). Data from the Add Health cohort study showed that if all participants had been breastfed for 3 months, the gradient in biomarker-measured chronic inflammation, related to higher levels of inflammatory cytokines in adipose tissue among participants who were obese in adulthood, would have been reduced by 80% (41). Investing in improving breastfeeding rates can significantly reduce the intergenerational transmission of health disparities and needs to be a more important public health priority in the United States.

This study provides a national benchmark for future epidemiologic monitoring of breastfeeding rates. Increasing breastfeeding initiation and duration in the United States will require an integrated and multifaceted approach by that includes healthcare quality improvement, public policy and educational system interventions (including in high schools) to provide clear and consistent information on breastfeeding benefits for mothers, infants, and future generations. In addition to reimbursement and workforce pipelines for breastfeeding peer support and lactation consultants, additional community resources are needed to influence the knowledge and attitudes of younger, lower income populations before pregnancy. The fact that over 75% of college educated PRAMS respondents were breastfeeding at 6 months or time of survey provides a benchmark for what is possible for all new birthing parents.

## Data availability statement

The original contributions presented in the study are included in the article/supplementary materials, further inquiries can be directed to the corresponding author/s.

## Ethics statement

The requirement of ethical approval was waived as the study is IRB-exempt because it uses publicly available, deindentified data. The studies were conducted in accordance with the local legislation and institutional requirements. The participants provided their written informed consent to participate in this study.

## Author contributions

LD: Conceptualization, Writing – original draft. LY: Conceptualization, Writing – review & editing. JF: Conceptualization, Writing – review & editing, Data curation, Formal analysis, Methodology.
